# INFRAFRONTIER: a European resource for studying the functional basis of human disease

**DOI:** 10.1007/s00335-016-9642-y

**Published:** 2016-06-04

**Authors:** Michael Raess, Ana Ambrosio de Castro, Valérie Gailus-Durner, Sabine Fessele, Martin Hrabě de Angelis, the INFRAFRONTIER Consortium

**Affiliations:** INFRAFRONTIER GmbH, 85764 Neuherberg, Germany; Institute of Experimental Genetics & German Mouse Clinic, Helmholtz Zentrum München, 85764 Neuherberg, Germany

## Abstract

Ageing research and more generally the study of the functional basis of human diseases profit enormously from the large-scale approaches and resources in mouse functional genomics: systematic targeted mutation of the mouse genome, systemic phenotyping in mouse clinics, and the archiving and distribution of the mouse resources in public repositories. *INFRAFRONTIER, the European research infrastructure for the development, systemic phenotyping, archiving and distribution of mammalian models*, offers access to sustainable mouse resources for biomedical research. INFRAFRONTIER promotes the global sharing of high-quality resources and data and thus contributes to data reproducibility and animal welfare. INFRAFRONTIER puts great effort into international standardisation and quality control and into technology development to improve and expand experimental protocols, reduce the use of animals in research and increase the reproducibility of results. In concert with the research community and the *International Mouse Phenotyping Consortium (IMPC)*, INFRAFRONTIER is currently developing new pilot platforms and services for the research on ageing and age-related diseases.

## Comprehensive analysis of ageing phenotypes in a mouse clinic

Neff et al. (2013) recently reported on a large-scale study to test whether the extended lifespans of mice treated with the drug rapamycin (Harrison et al. 2009; Miller et al. 2011) can be attributed to direct rapamycin effects on ageing. They used the unique setup of the *German Mouse Clinic* (www.mouseclinic.de), which allows to comprehensively study the effects of genotypes, environmental factors or treatments on phenotypes (Fuchs et al. 2012; Gailus-Durner et al. 2005, 2009), to carry out a comprehensive analysis of rapamycin effects on more than 150 ageing phenotypes across 25 different tissues in male C57BL/6 J mice. The study, which was carried out in cooperation with the German Center for Neurological Diseases (DZNE), analysed three cohorts of mice on rapamycin supplemented diets starting in early adulthood (4 months), midlife (13 months) and late in life (20–22 months). Each cohort was treated for at least 12 months before a comprehensive phenotypic analysis was performed (starting at 16, 25 and 34 months of age, respectively). Those ageing phenotypes rescued by rapamycin were further assessed in groups of young adult mice to identify ageing-independent rapamycin effects.

As in previous studies, Neff et al. (2013) observed that rapamycin extended lifespan in the treated mice. There was, however, no systematic effect of the drug on ageing phenotypes, since only a subset of traits was rescued by rapamycin. Moreover, many of those traits were equally affected by rapamycin in young mice, suggesting ageing-independent drug effects. The authors therefore conclude that the effects of rapamycin on longevity are not necessarily mediated by direct effects on ageing traits but could rather be the results of a reduction of life-limiting pathologies such as specific cancers by rapamycin.

The study by Neff and colleagues exemplifies the strength of the mouse clinic concept, which allows obtaining a truly systemic understanding of how disease phenotypes are affected by the genetic make-up, lifestyle or treatment regime (or a combination of those). Researchers who want to test their hypothesis on how ageing is affected by specific treatments or challenges can plan, design and initiate the experiments in their own facilities, where the setup of the cohorts, breeding and ageing can be done. The comprehensive phenotypic analysis is then carried out by the mouse clinic, and researchers profit from the extensive expert knowledge, sophisticated high-end technology and tailor-made statistical analysis offered by these large-scale phenotyping facilities. European mouse clinics offering a standardised first-line phenotyping screen are located in Germany (German Mouse Clinic, Munich), France (Institute Clinique de la Souris, Strasbourg) and the United Kingdom (Mary-Lyon Centre, Harwell and Wellcome Trust Sanger Institute, Hinxton). A novel mouse clinic is currently becoming operative in the Czech Republic (Czech Centre for Phenogenomics, Prague). All these European mouse clinics are organised in the *INFRAFRONTIER* network (www.infrafrontier.eu, see below). Modelled on these successful examples, mouse clinics meanwhile also operate in North America, Asia and Australia.

## European large-scale functional genomics initiatives and the INFRAFRONTIER research infrastructure

Mouse studies like the one of Neff and colleagues help discover the functional basis of ageing-related diseases and other human disease conditions (Fuchs et al. 2011; Schughart et al. 2012; Vandamme 2015) and are also important in preclinical screening (Varga et al. 2010) and drug development (Neschen et al. 2015; Sharpless and Depinho 2006; Zambrowicz and Sands 2003). As exemplified by the mouse clinic concept, Europe has played a leading role, both in the initiation of large-scale approaches for functional mouse genomics and in the continuous cross-laboratory standardisation of operation procedures and quality measures that allowed them to evolve into truly global scientific enterprises (Beckers et al. 2009; Rosenthal and Brown 2007).

Using a combination of gene trapping and gene targeting the *European Conditional Mouse Mutagenesis Program* (EUCOMM) as part of the *International Knock*-*Out Mouse Consortium* (IKMC) created a resource of mutated ES cells and mice for each protein-coding gene in the mammalian genome (Collins et al. 2007; Friedel et al. 2007). Drawing on this resource, a consortium of European mouse clinics participating in the EUMODIC initiative (*European Mouse Disease Clinic*) showed that it is feasible and also scalable to carry out a multi-laboratory programme for the broad-based phenotypic analysis of mammalian gene function (Angelis et al. 2015; Morgan et al. 2010). The success of this programme led to the initiation of the International Mouse Phenotyping Consortium (IMPC, www.mousephenotype.org), which brought large-scale phenotyping to the global level, with the aim to create an openly accessible catalogue of mammalian gene function (Brown and Moore 2012; Koscielny et al. 2014).

**Fig. 1:**
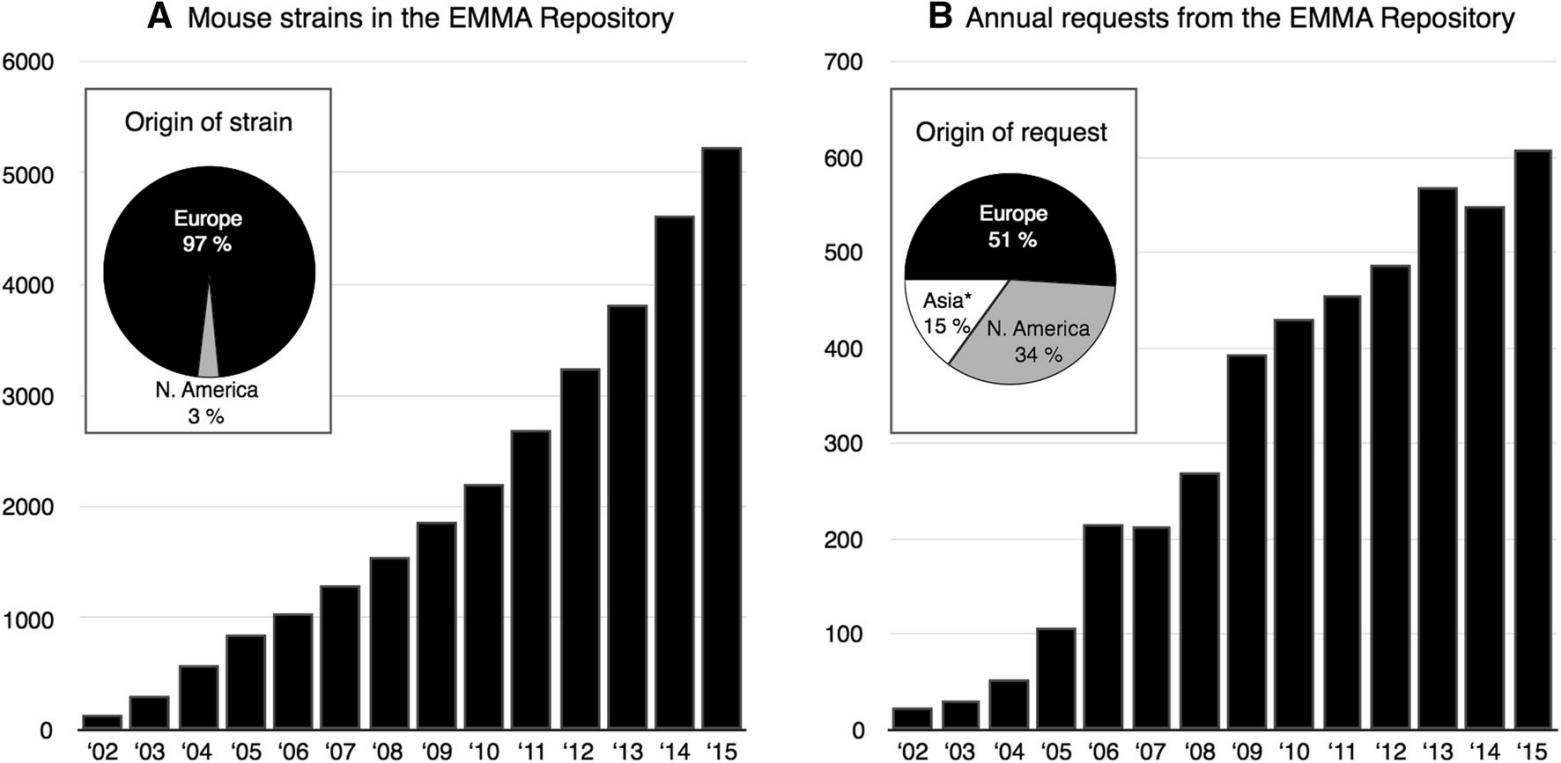
**a** The European Mouse Mutant Archive (EMMA) as part of the INFRAFRONTIER Research Infrastructure is worldwide the third largest public repository of mutant mouse strains, with more than 5000 strains available. **b** The number of yearly requests for mouse strains in the EMMA repository is continuously growing. Almost half of the requests come from outside Europe. Mouse lines deposited by the large-scale initiatives IKMC/IPMC are in high demand and constituted approximately 50 % of all requests in 2015. *Asterisk* Including requests from Oceania and Australia

At the same time as the large-scale approaches for the mutagenesis and systemic phenotyping of mice were developed, a consortium of European research centres started to build the European Mouse Mutant Archive (EMMA), a repository that archives and distributes mutant mouse lines submitted by individual researchers and generated by the large-scale initiatives IKMC and IMPC (Hagn et al. 2007). Starting in 2001, the EMMA repository has been continually growing (Fig. 1a) and currently is the third largest repository for mouse strains listed on the *International Mouse Strain Resource* (www.findmice.org/repository) (Eppig et al. 2015), distributing mouse strains to biomedical researchers around the globe (Fig. 1b).

**Fig. 2:**
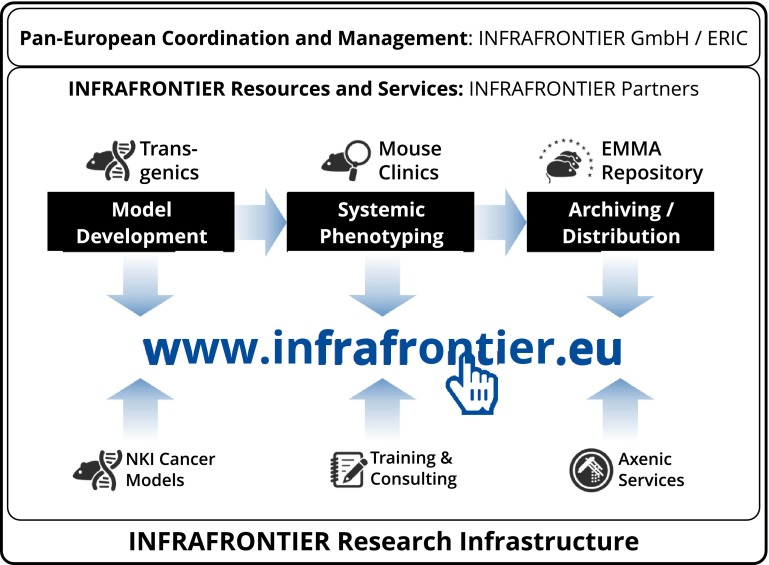
The resources and services of the INFRAFRONTIER Research Infrastructure can be accessed at the central web portal. They are provided by the European INFRAFRONTIER partners and centrally coordinated by the INFRAFRONTIER GmbH/ERIC (see text for further explanations)

Building sufficient capacities and securing sustainable access to high-quality resources for mouse functional genomics across national borders is a challenge that requires novel funding and governance models (Mishra et al. 2016). In Europe, therefore, a consortium of 23 research centres across 15 countries established the pan-European *INFRAFRONTIER Research Infrastructure for the generation, systemic phenotyping, archiving and distribution of mouse disease models*. The INFRAFRONTIER partners are mouse clinics and EMMA archiving and distribution centres. They offer an integrated portfolio of resources and services that can be accessed via a central web portal (www.infrafrontier.eu, Meehan et al. 2015) (Fig. 2). In 2015, more than 1300 user projects were handled by the INFRAFRONTIER Research Infrastructure.

The strategic importance of having available a sustainable resource for functional mouse genomics in Europe was underlined by the inclusion of INFRAFRONTIER on the first Roadmap of the *European Strategy Forum on Research Infrastructures* (ESFRI) issued in 2006 (www.esfri.eu). This document lists those pan-European research infrastructures that are crucial for building the European research area (ERA) and keeping a competitive edge to European science. According to the ESFRI definition, research infrastructures “*are facilities, resources or services of a unique nature that have been identified by European research communities to conduct top*-*level activities in all fields. This…covers major equipment or sets of instruments, in addition to knowledge*-*containing resources such as collections, archives and data banks*”. ESFRI also recognised that a sustainable pan-European organisation and governance is required to efficiently manage capacities and access to resources and services, particularly if the research infrastructure is distributed across several European countries. Therefore, in 2013 the INFRAFRONTIER GmbH was established to act as the central management unit of the INFRAFRONTIER Research Infrastructure. Meanwhile the process has been initiated to transform the INFRAFRONTIER GmbH into a *European Research Infrastructure Consortium (ERIC), a legal form particularly created by the European Commission for pan*-*European research infrastructures like INFRAFRONTIER.* In the fourth iteration of the ESFRI Roadmap which has been recently published, the INFRAFRONTIER Research Infrastructure is highlighted as one of the ‘Landmark Projects’.

## INFRAFRONTIER helps improve animal welfare and data reproducibility

Biomedical research infrastructures like INFRAFRONTIER play a key role in preserving and sharing resources and data created by publicly funded research (Mishra et al. 2016). By offering open access to centralised high-quality resources, they help prevent duplication of efforts and wasting of public resources. By setting standard operation procedures and strict quality control measures, they help reduce the irreproducibility of scientific results in preclinical research, the costs of which have been recently estimated to be 28 billion $ per year in the US alone (Freedman et al. 2015).

In the case of the INFRAFRONTIER Research Infrastructure, this has direct implications on animal welfare and on the validity of scientific results based on studies in mice, which often suffer from poorly applied scientific standards (Perrin 2014; Justice and Dhillon 2016). The INFRAFRONTIER partners are strongly committed to the implementation of the 3R’s principle (*replacement, reduction, refinement*; Sabaté 2015) and do endorse the ARRIVE (Animal Research: Reporting of In Vivo Experiments) Guidelines which are promoted by the *National Centre for Replacement, Refinement & Reduction of animals in Research* (www.nc3rs.org.uk/ARRIVE). Applying and reporting strict scientific quality standards is even more important with the spread of novel gene editing technologies like CRISPR/Cas9. Gene editing may make mouse mutant generation more generally accessible, but experience in mouse genetics is required to take into account the effects of genetic background, additional mutations and husbandry conditions such as housing, diet or the gut microbiome (Ericsson et al. 2015; Lloyd et al. 2015). Moreover, genotypes may get lost due to breeding errors or fatal diseases in the mouse colony. More than once experimental results could not be reproduced because the genotype could not be maintained. Public repositories such as INFRAFRONTIER/EMMA have both the experience and the quality measures in place to safeguard scientifically valuable mouse strains.

The INFRAFRONTIER Research Infrastructure and its international partners put a lot of effort into the development of new technologies that help reduce animal numbers and refine existing animal experiments. Improving quality control by means of blastocyst genotyping reduces the number of animals required for cryopreservation, as well as processing time, technical requirements and costs (Scavizzi et al. 2015). Moreover, the methods described can also be applied to optimise experimental design when using CRISPR/Cas9 technologies in mouse model generation. Improving in vitro *fertilisation* technology and using refined cryoprotective agents is another way to reduce animal numbers and overall costs (Guan et al. 2014). INFRAFRONTIER technology development also focuses on the improvement of transportation procedures of mouse sperm and embryos (Kenyon et al. 2014). Coupled with training activities to enable INFRAFRONTIER users to work with frozen material, these activities have helped to markedly reduce the need for shipping live animals from the EMMA repository.

Overall, the INFRAFRONTIER Research Infrastructure has established a successful model “for sharing animal models, protocols and genetic information, as well as a wealth of cross-referenced data”, and promotes transparency in animal research, as has been recently recognised by McGrath et al. (2015).

## Perspectives for ageing research

Ageing research addresses a major societal challenge. As life expectancy increases globally, also the related health care costs for age-related diseases surge. Publicly funded research infrastructures have a public service mission to address these challenges. The INFRAFRONTIER Research Infrastructure does this in several ways: On the strategic level, INFRAFRONTIER engages with European programmes addressing ageing and age-related diseases, such as the joint programming initiatives “More Years, Better Lives” or the “EU Joint Programme—Neurodegenerative Disease Research (JPND)”. On the scientific level, INFRAFRONTIER currently interfaces with the European ageing community to develop pilot platforms and services for ageing research that can be offered at the European level. These activities are tightly integrated with the current efforts of the International Mouse Phenotyping Consortium to set up a standardised ageing pipeline. The INFRAFRONTIER Research Infrastructure, in concert with the global large-scale resources for mouse functional genomics, already has a lot to offer to the biomedical research community for ageing, and it will put a lot of effort into expanding this even further in the coming years.
